# Population pharmacokinetic properties of artemisinin in healthy male Vietnamese volunteers

**DOI:** 10.1186/s12936-016-1134-8

**Published:** 2016-02-16

**Authors:** Sofia Birgersson, Pham Van Toi, Nguyen Thanh Truong, Nguyen Thi Dung, Michael Ashton, Tran Tinh Hien, Angela Abelö, Joel Tarning

**Affiliations:** Unit for Pharmacokinetics and Drug Metabolism, Department of Pharmacology, University of Gothenburg, Gothenburg, Sweden; Oxford University Clinical Research Unit, South East Asia Infectious Disease Clinical Research Network, Hospital for Tropical Diseases, Ho Chi Minh City, Vietnam; Hospital for Tropical Diseases, Ho Chi Minh City, Vietnam; Centre for Tropical Medicine and Global Health, Nuffield Department of Clinical Medicine, University of Oxford, Oxford, UK; Mahidol-Oxford Tropical Medicine Research Unit, Faculty of Tropical Medicine, Mahidol University, Bangkok, Thailand

## Abstract

**Background:**

Artemisinin-based combination therapy is recommended as first-line anti-malarial treatment worldwide. A combination of artemisinin with the long acting drug piperaquine has shown high efficacy and tolerability in patients with uncomplicated *Plasmodium falciparum* infections. The aim of this study was to characterize the population pharmacokinetic properties of artemisinin in healthy male Vietnamese volunteers after two different dose sizes, formulations and in a combination with piperaquine. A secondary aim was to compare two different methods for the evaluation of bioequivalence of the formulations.

**Methods:**

Fifteen subjects received four different dose regimens of a single dose of artemisinin as a conventional formulation (160 and 500 mg) and as a micronized test formulation (160 mg alone and in combination with piperaquine phosphate, 360 mg) with a washout period of 3 weeks between each period (i.e. four-way cross-over). Venous plasma samples were collected frequently up to 12 h after dose in each period. Artemisinin was quantified in plasma using liquid chromatography coupled with tandem mass spectrometry. A nonlinear mixed-effects modelling approach was utilized to evaluate the population pharmacokinetic properties of the drug and to investigate the clinical impact of different formulations.

**Results:**

The plasma concentration–time profiles for artemisinin were adequately described by a transit-absorption model with a one-compartment disposition, in all four sequences simultaneously. The mean oral clearance, volume of distribution and terminal elimination half-life was 417 L/h, 1210 L and 1.93 h, respectively. Influence of formulation, dose and possible interaction of piperaquine was evaluated as categorical covariates in full covariate approaches. No clinically significant differences between formulations were shown which was in accordance with the previous results using a non-compartmental bioequivalence approach.

**Conclusions:**

This is the first population pharmacokinetic characterization of artemisinin in healthy volunteers. Increasing the dose resulted in a significant increase in the mean transit-time but the micronized formulation or concomitant piperaquine administration did not affect the pharmacokinetic properties of artemisinin. The results from the traditional bioequivalence evaluation were comparable with results obtained from mixed-effects modelling.

## Background

Malaria is still a major health problem and the emergence of multidrug-resistant *Plasmodium falciparum* parasites has further diminished the efficacy of available drugs [1]. Artemisinin-based combination therapy (ACT) is recommended by the World Health Organization as first-line treatment of uncomplicated *P.falciparum* malaria. However, several reports indicate emerging artemisinin-resistance in Southeast Asia characterized by increased parasite clearance times for artemisinin derivatives in patients with falciparum malaria [2–6]. A recent study has also identified a molecular marker, the K13-propeller gene mutations, associated with artemisinin resistance [7]. Despite this emerging resistance, artemisinin and its derivatives are still effective in Africa and most regions of Southeast Asia. Treatment failure is commonly less than 5 % at day 28 when administered in a combination with a longer acting anti-malarial drug in falciparum malaria [8–12]. However, alarming results have recently demonstrated substantially decreased therapeutic efficacy of dihydroartemisinin–piperaquine in Western Cambodia [12, 13].

Artemisinin has previously been used as monotherapy (5-day treatment) with rapid parasite clearance, although with a high recrudescence rate due to the short half-life (1.4–2.6 h) [14–16]. Increasing the dose schedule to a 7-day treatment did not substantially decrease recrudescence [14]. Due to the pronounced metabolic auto-induction of artemisinin it has not been commonly used in artemisinin-based combinations. Studies in both patients and healthy volunteers have shown a decrease of 70–80 % to the exposure of artemisinin from the first day of dosing to the seventh day of dosing [17–19]. This reduction is a result of induction of several enzymes in the cytochrome P450 (CYP) family where CYP2B6 is the main metabolizing enzyme of artemisinin with minor contribution of CYP2A6 and CYP3A4 [20–23]. Repeated oral administration of artemisinin over 7 days has shown a two-fold increase in CYP2C19 and CYP2B6 activity at day 7 compared to un-induced levels at day 1 [21, 22]. Significantly increased activity in CYP2C19, CYP3A and CYP1A2 has also been reported after repeated administration of artemisinin [20].

Piperaquine is a long-acting drug for co-administration in ACT, responsible for killing residual parasites and preventing recrudescence. The fixed combination of dihydroartemisinin and piperaquine is well tolerated with high cure rates in areas with multidrug resistant *P. falciparum* [24–26].

Artemisinin and piperaquine, given as a combination, has shown excellent parasite clearance and fever clearance times, comparable to that of dihydroartemisinin–piperaquine and mefloquine–artesunate [27–29]. These three combinations all resulted in 100 % cure rates with no recrudescent malaria at day 28. However, there was a lower incidence of adverse events in the gastrointestinal tract after administration of artemisinin–piperaquine as compared to after administration of dihydroartemisinin–piperaquine. Artemisinin–piperaquine could also possibly be implemented as a simplified dosing scheme of a 2-day therapy, compared to 3 days in commonly used ACT. This was evaluated in the work by Thanh et al. [30] where a 2-day therapy of artemisinin–piperaquine was compared to the conventional 3-day treatment of artesunate–amodiaquine. Both treatments had similar effectiveness but the artesunate–amodiaquine combination had faster parasite clearance times. However, this shorter treatment course raises concerns on parasite resistance development and may not be a suitable alternative to the traditional 3-day treatment.

There are no studies presented on the pharmacokinetics of intravenous administration of artemisinin, and no absolute oral bioavailability has been established. However, following intramuscular oil solution or suppositories the relative oral bioavailability was approximately 30 % [17, 31]. The low bioavailability of oral administration of artemisinin is probably due to low solubility and/or high first pass metabolism [31, 32]. It is, therefore, desirable to increase the bioavailability of artemisinin. Attempts have been made to increase the oral bioavailability by adding β- and γ-cyclodextrin complexes and thereby increasing artemisinin solubility [33]. Both the in vivo rate and extent of artemisinin absorption was found to increase compared to the reference substance. Another alternative would be to re-formulate artemisinin as a micronized formulation to increase solubility and thereby bioavailability [34].

There have only been two previous studies successfully describing the population pharmacokinetics of artemisinin by Sidhu et al. [35] and by Batty et al. [36]. In the study by Sidhu et al. only a sparse sampling schedule was applied in paediatric and adult patients with falciparum malaria, limiting its potential to adequately describe the pharmacokinetics and to identify relevant covariates. Batty et al. developed a population pharmacokinetic model for three groups of paediatric patients. They concluded that the pharmacokinetic parameters of artemisinin in children are comparable to those in adults.

The objective of this study was to characterize the pharmacokinetic properties of artemisinin in healthy Vietnamese volunteers after different doses, formulations and in combination with piperaquine. The frequent sampling schedule allowed for investigation of both absorption properties and possible biphasic disposition of artemisinin. A secondary aim was to compare the traditional model-independent bioequivalence methodology to a modelling-based approach.

## Methods

### Study design and ethical approval

The pharmacokinetic study was conducted at the Hospital for Tropical Diseases in Ho Chi Minh City (HTD-HCMC), Vietnam. Clinical details and non-compartmental analysis results are reported in full elsewhere [23]. The investigation was a single-centre, single-dose, open-label, randomized, four-sequence, cross-over study with a 3-week washout period (i.e. >5 artemisinin half-lives) between occasions. The clinical trial protocol was approved by the internal Scientific and Ethical Committee of the HTD-HCMC and the Oxford Tropical Research Ethics Committee (OxTREC 019-06), University of Oxford, Oxford, UK. The volunteers were randomly assigned to 1 of the 24 possible sequences for the 4 treatments.

Treatment 1 (T1) comprised a single dose of two hard gelatin capsules [Trademark: Coni-Snap Capsules (Capsugel [Thailand] Co., Ltd., Ayutthaya, Thailand)] (size 2), each containing 80 mg of micronized artemisinin powder; treatment 2 (T2) comprised a single dose of two hard gelatin capsules, each containing 80 mg of artemisinin powder (reference Vietnamese low-dose formulation); treatment 3 (T3) comprised a single dose of two hard gelatin capsules, each containing 250 mg of artemisinin powder (reference Vietnamese dose-strength formulation); and treatment 4 (T4) comprised a single dose of two tablets, each containing 80 mg of micronized artemisinin and 360 mg of piperaquine phosphate.

Tolerability was assessed daily by the clinician during each study occasion (using an adverse-event report form) and at the follow-up visit 1 week after last study occasion (using an adverse-event report form and laboratory assessment). Adverse events were accessed by using an open question about potential health problems during the study and followed up with a questionnaire if any health problems had occurred since the last consultation.

### Study subjects

Study design and possible adverse drug effects were explained to all volunteers in their own language before initiation. Volunteers providing written informed consent were considered for enrollment. Clinical and laboratory screenings were performed at HTD-HCMC, Vietnam, and the results were evaluated before enrollment. Clinical evaluation and laboratory assessment (full haematology and biochemistry) were performed at follow-up 1 week after the last study visit.

### Exclusion criteria

Intake of any anti-malarial agent during the previous 3 months, participation in an ongoing clinical drug study or within the last 3 months, involvement in the planning and/or conduct of the study, inability to comply with study procedure during the 10 weeks of participation, or intending to donate blood within 6 months after study start. Healthy volunteers, according to clinical and laboratory screening data, who fulfilled all of the inclusion but none of the exclusion criteria were enrolled in the study.

### Blood sampling

An intravenous indwelling cannula was introduced and maintained in the antecubital vein and kept for 12 h during blood sample collection at each study visit. A blood volume of 0.5 mL was discarded before sample collection to avoid drug dilution effects. A 5 mL blood sample was drawn into lithium heparin tubes (Hong Thien My Medical Equipment Joint Stock Co., Ho Chi Minh City, Vietnam) before drug administration (pre-dose) and at the following times after drug administration (post-dose): 0.25, 0.5, 1, 1.5, 2, 2.5, 3, 4, 5, 6, 7, 8, 10, and 12 h. The same time schedule was applied for all study visits. Saline solution (0.9 % sodium chloride, 2 mL) was used to flush the cannula after blood collection. Blood samples were immediately centrifuged at 3000×*g* for 10 min at 20 °C. Plasma were transferred to cryotubes within 10 min after centrifugation and stored at –70 °C. All samples were freighted on dry ice to the Department of Clinical Pharmacology at Mahidol–Oxford Tropical Medicine Research Unit (Bangkok, Thailand) where the plasma samples were analysed. The laboratory is a participant in the QA/QC proficiency testing programme supported by the Worldwide Antimalarial Resistance Network (WWARN) [37].

### Drug analysis

Plasma concentrations of artemisinin were determined using a previously published LC–MS/MS method performed on an API 5000 system (Applied Biosystems/MDS SCIEX, Foster City, California) [38]. The limit of detection was 0.257 ng/mL with a linear range of quantification of 1.03–762 ng/mL. Three independent quality-control samples in plasma (2.89, 40.7, and 571 ng/mL) were prepared freshly and analysed in triplicates together with each batch of extracted plasma samples. The coefficient of variation was below 5 % for all quality control samples.

### Pharmacokinetic analysis

Artemisinin plasma concentrations were transformed into their natural logarithms and concentration–time data characterized using nonlinear mixed-effects modelling in the NONMEM software (version 7.1.2; ICON Development Solutions, MD). Post-processing and automation were performed using Pearl-Speaks-NONMEM (PsN) (version 3.4.2) [39]; Pirana (2.4.0) [40], and Xpose (version 4.0) [41] package in R (version 2.13.1; The R Foundation for Statistical Computing). The first-order conditional estimation method with interactions was used throughout the modelling. Model discrimination was based on the objective function value (OFV; computed by NONMEM as proportional to minus twice the log likelihood of the data) and basic goodness-of-fit graphical analysis. A drop of 3.84 was considered significant (*p* < 0.05) when comparing two nested models with one degree of freedom difference. Only 5 % of plasma samples (all within 30 min of dosing) were measured to be below the limit of quantification and therefore omitted in the analysis. One-, two- and three-compartment models with first-order elimination from the central compartment were fitted to the plasma concentration–time data for artemisinin. Zero- and first-order absorption with and without an absorption lag-time was evaluated. A sequential absorption model (zero- and first-order) and a transit compartment model with 1–10 fixed transit compartments were also tried [42]. A relative bioavailability parameter, fixed to unity for the population, was evaluated to allow inter-individual variability in the absorption of artemisinin.

Inter-individual variability was added exponentially as illustrated for clearance (Eq. 1).

1 $$CL/F_{i} = TV(CL/F)*\exp (\eta_{i,CL/F} )$$

where CL/F_i_ is the oral elimination clearance individually estimated for the *i*th patient and TV(CL/F) is the typical clearance value for the population. η_i,CL/F_ is the inter-individual variability, assumed to be normally distributed around zero and with a variance ω^2^. Inter-occasion variability was evaluated on all parameters. The residual random variability was modeled with an additive error model on the log-transformed drug concentrations, being essentially equivalent to an exponential residual error on an arithmetic scale.

### Covariates

After graphical evaluation relevant covariates were chosen for the automatic covariate analysis. The continuous covariates were age, weight, systolic blood pressure, diastolic blood pressure, heart rate and biochemical and haematological measurements (i.e. urea, glucose, creatinine, total bilirubin, total protein, albumin, globulin, magnesium, ferritin, ALAT, ASAT, leukocytes, albumin/globulin ratio, ferritin, erythrocytes, haemoglobin, haematocrit, reticulocytes, thrombocytes, neutrophils, eosinophils, monocytes, basophils, lymphocytes). Smoking, formulation, dose size and possible drug–drug interactions with concomitant piperaquine administration were investigated as categorical covariates.

Stepwise forward inclusion (*p* < 0.05) were used for both continuous and categorical covariates followed by a stepwise backward exclusion (*p* < 0.01). The covariates were tested with a linear, hockey-stick, exponential and a power relationship. Bodyweight, centered on the median weight of the population, was also tested as an allometric function on clearance and volume parameters, where clearance were scaled to mass to a power of 0.75 and where the volume was scaled to mass to the power of one [43–45].

Formulation effect, dose effect and possible drug–drug interactions with concomitant piperaquine administration were also investigated in the final pharmacokinetic model using a full covariate approach i.e. the covariate of interest was added as a categorical covariate on all estimated fixed effects simultaneously. Formulation effect and dose effect were added to mean transit time and relative bioavailability while the drug–drug interaction with concomitant piperaquine was added to mean transit time, clearance and volume of distribution. These three covariate models were bootstrapped (n = 500) and the 90 % confidence interval of the covariate effects calculated to investigate the impact of each covariate on the pharmacokinetic properties of artemisinin. A covariate related change in the parameter estimate of more than 20 % was assumed to be of clinical relevance.

### Model evaluation

Basic goodness-of-fit characteristics were evaluated by plotting observed drug concentrations against individually predicted and population predicted drug concentrations and by plotting conditionally weighted residuals against population predicted drug concentrations and time [46]. Eta and epsilon shrinkage were calculated to evaluate the reliability of the goodness-of-fit diagnostics [47]. Visual predictive checks were performed using 2000 simulations at each concentration time point (protocol time points were used for binning). Bootstrap diagnostics (1000 re-sampled datasets stratified on formulation) were performed for the final model to obtain standard errors for parameter estimates and non-parametric confidence intervals around these parameters.

### Simulations

Mean concentration–time profiles for healthy volunteers (present study), adult patients [35] and paediatric patients [36] were simulated in the software Berkeley Madonna [48]. Pharmacokinetic parameters were implemented as described in literature and weighted-adjusted doses were implemented identical to that given to healthy volunteers.

## Results

**Fig. 1:**
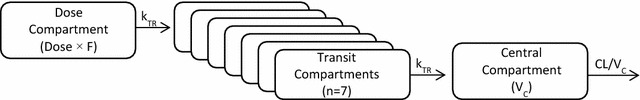
Structural representation of the final model describing artemisinin population pharmacokinetics in healthy male Vietnamese subjects. k_TR_, absorption rate constant; CL, elimination clearance; V_C_, volume of distribution of the central compartment; F, relative oral bioavailability

Fifteen healthy Vietnamese male volunteers aged 19–41 years were enrolled and completed the study. Full demographic characteristics are given in Table 1. Seven hundred eighty-six (786) plasma samples of artemisinin were used in the pharmacokinetic analysis. Data from all four regimens were successfully modeled simultaneously with a nonlinear mixed-effects approach. The pharmacokinetics of artemisinin was best characterized by a one-compartment disposition model with seven transit compartments in the absorption phase (Fig. 1). When the model was extended from one to two distribution compartments, the OFV decreased significantly (ΔOFV = −16.9). However, this resulted in a substantial increase in the peripheral volume of distribution (i.e. 66,900 L) leading to an unrealistic value for the terminal elimination half-life (127 h) and was not carried forward. The addition of a third compartment did not improve the model fit significantly (*p* > 0.05). A transit-compartment absorption model (n = 7) was significantly better than all other absorption models tested (ΔOFV = −134). Inter-occasion variability on clearance and mean transit-time improved the model fit significantly (ΔOFV = −178). Inter-individual variability was retained on relative bioavailability with no additional benefit of implementing inter-individual variability on clearance, mean transit time or volume parameters. The population-derived pharmacokinetic estimates with relative standard errors are presented in Tables 2 and 3.

**Table 1:** Demographic data at enrollment for the 15 healthy male Vietnamese subjects

Parameter	Mean (SD)	Median [range]
Age (years)	28.1 (8.5)	23 [19–41]
Weight (kg)	59.0 (9.3)	58 [43–80]
Height (cm)	166 (6.4)	167 [158–180]
Systolic blood pressure (mmHg)	115 (7.4)	110 [100–130]
Diastolic blood pressure (mmHg)	64.0 (6.3)	60 [60–80]
Heart rate (beats/min)	80.3 (4.2)	80 [70–86]

*SD* standard deviation

**Table 2:** Parameter estimates of the final model describing artemisinin population pharmacokinetics in healthy male Vietnamese subjects

Parameter	Population estimate (RSE %)	95 % CI	IIV/IOV* CV % (RSE %)	95 % CI
CL/F (L/h)	417 (9.32)	350-501	17.1* (34.3)	11.1-22.6
V/F (L)	1210 (9.02)	1030-1450	–	–
Nr. trans comp	7 *fix*	–	–	–
MTT (h)	0.787 (5.97)	0.702-0.891	53.9* (20.3)	41.6-66.9
F (%)	100 (fixed)	–	34.3 (52.3)	17.3-50.5
σ (CV %)	51.6 (5.84)	44.9-58.1	–	–

CL/F, apparent elimination clearance; V/F, apparent volume of distribution; Nr. trans comp, number of transit compartments in the absorption model; MTT, mean transit-time of the absorption phase; F, relative oral bioavailability; σ, additive residual error. RSE is the relative standard error calculated as 100 × *standard deviation*/*mean*. CV % is the coefficient of variation calculated as $100 \times SQRT\left( {e^{variance} - 1} \right)$ for inter-individual variability (IIV) and inter-occasion variability (IOV). 95 % CI, 95 % confidence intervals calculated as the 2.5 and 97.5 percentiles of bootstrap estimates. Parameter estimates are based on population mean values from NONMEM, RSE % and CI values are based on 954 successful bootstrap runs (out of 1000)

**Table 3:** Secondary parameters of the final model describing artemisinin population pharmacokinetics in healthy male Vietnamese subjects

Parameter	Treatment 1	Treatment 2	Treatment 3	Treatment 4
C_max_ (ng/mL)	111 [45.2–183]	96.7 [52.1–169]	244 [133–479]	144 [58–200]
T_max_ (h)	1.41 [0.762–2.06]	1.09 [0.773–2.28]	1.72 [1.12–3.65]	0.992 [0.628–1.90]
t_1/2_ (h)	1.97 [1.64–3.37]	1.80 [1.46–3.20]	1.93 [1.71–2.43]	2.02 [1.64–2.42]
AUC_0–12_ (ng × h/mL)	461 [144–651]	342 [178–624]	956 [462–1973]	462 [189–744]
AUC_∞_ (ng × h/mL)	441 [472–146]	349 [181–642]	994 [468–2040]	467 [192–761]

Secondary parameters estimated from the final model and values are presented as median [range]. *C*_*max*_ is the maximum concentration and *T*_*max*_ is the time to reach C_max_. *t*_*1/2*_ is the estimated terminal elimination half-life. *AUC*_*0*–*12*_ is the accumulated area under the concentration–time curve from time zero to 12 h after dose and *AUC*_*∞*_ is the accumulated area under the concentration–time curve from time zero extrapolated to infinity. Treatment 1 was administrated as 160 mg micronized artemisinin, treatment 2 was 160 mg of the reference formulation of artemisinin, treatment 3 was 500 mg of the reference formulation of artemisinin and treatment 4 was 160 mg micronized artemisinin and 720 mg of piperaquine phosphate

Bodyweight, as an allometric function, did not improve the model fit and was not retained in the final model. Haemoglobin levels on mean transit-time and eosinophils counts on volume of distribution were both significant in the stepwise covariate selection. However, the reduction in the inter-individual variability of these parameters and the residual error was minimal. Furthermore, no improvement of goodness of fit diagnostics was seen after the addition of these covariates. Consequently, they were rejected in the final model. No other tested covariates were significant in the stepwise covariate approach.

**Fig. 2:**
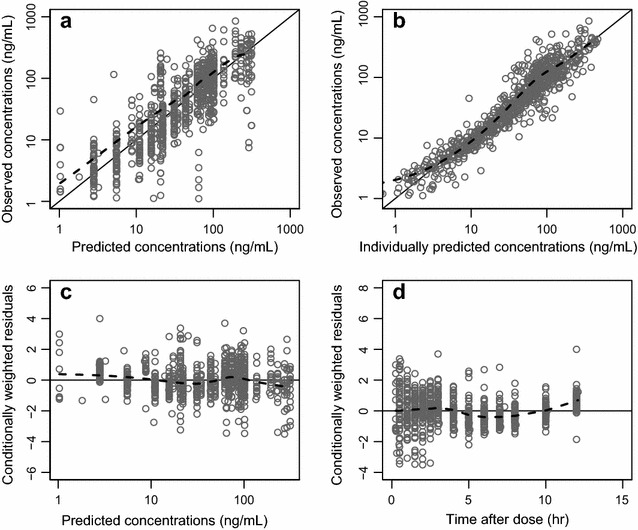
Goodness-of-fit diagnostics of the final model describing artemisinin population pharmacokinetics in healthy male Vietnamese subjects. *Open circles* represent observed concentrations versus population predicted concentrations (**a**) and individually predicted concentrations (**b**), and conditionally weighted residuals versus population predicted concentrations (**c**) and time after dose (**d**). *Broken lines* are locally weighted least-squares regressions; *solid lines* represent the lines of identity. The concentrations are plotted on a logarithmic scale (base 10)

**Fig. 3:**
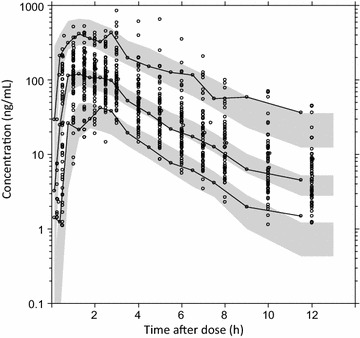
Prediction-corrected visual predictive check of the final model describing artemisinin population pharmacokinetics in healthy male Vietnamese subjects. *Open circles* represents the observations, the *solid lines* are 5th, 50th and 95th percentiles of the observations, and *shaded areas* represent the 95 % confidence interval of simulated 5th, 50th and 95th percentiles

The final model showed satisfactory goodness-of-fit diagnostics (Fig. 2) with estimated epsilon shrinkage and eta shrinkages of <15 %. The final model showed good predictive performance, as illustrated by the visual predictive check, resulting in 4.22 % (95 % CI 2.69–7.80 %) and 4.22 % (95 % CI 2.81–7.67 %) of artemisinin observations below and above the simulated 90 % prediction interval, respectively (Fig. 3).

**Fig. 4:**
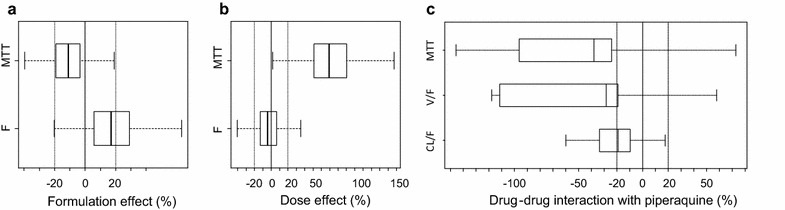
*Box* (25th and 75th percentile) and *whisker* (1.5 × interquartile range) plots of the full covariate models. The *solid black zero-line* represents no covariate effect (and the *dotted black lines* represent a covariate effect of ± 20 %). Formulation (**a**), dose (**b**) and the potential drug–drug interaction with piperaquine (**c**) were investigated as covariates. MTT is the mean transit-time, F is the relative oral bioavailability, V/F is the apparent volume of distribution and CL/F is the apparent elimination clearance. All covariates were added as categorical functions

In the full covariate model, mean transit-time increased by a median of 69.1 % with increasing dose size and there was a trend towards an increasing volume of distribution with increasing doses (160 vs 500 mg, Fig. 4b). None of the other parameters were influenced by artemisinin formulation (Fig. 4a), dose (Fig. 4b) or concomitant piperaquine administration (Fig. 4c).

**Fig. 5:**
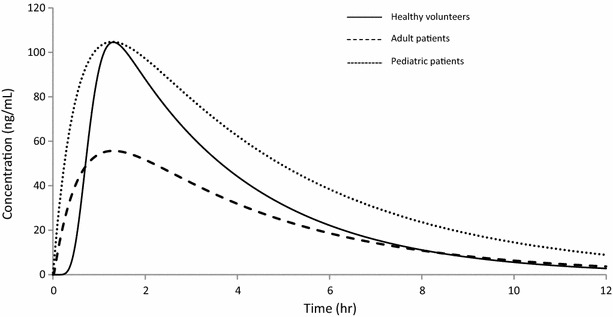
Simulated mean concentration–time profiles of healthy male Vietnamese volunteers (*solid line*, present study), adult patients (*dashed line*, [35]) and paediatric patients (*dotted line*, [36])

The simulations of mean concentration–time profiles for healthy volunteers, adult patients and paediatric patients are shown in Fig. 5.

## Discussion

In the fight against malaria, WHO states that the necessary tools are a combination of insecticide treated nets, indoor residual spraying, diagnostic tools and treatment with ACT [1]. However, the emerging resistance against the artemisinin compounds necessitates optimization of the current treatments to minimize the risk of developing drug resistance in areas where artemisinin resistance has not developed [2–4, 6, 49]. Artemisinin has not been used previously in ACT as a consequence of complicated pharmacokinetic properties due to the auto-induction of its own metabolizing CYP-enzymes [20–22]. However, in a short 2-day treatment the auto-induction will be less prominent [50] although with effectiveness comparable to a 3-day artesunate-mefloquine treatment [30].

In this work the pharmacokinetics of artemisinin was characterized using nonlinear mixed-effects modelling to estimate pharmacokinetic parameters and to evaluate the effect of different doses and a new micronized formulation. Artemisinin population pharmacokinetics has been reported in two previous publications [35, 36]. While previous studies were conducted in patients with falciparum malaria this was the first time a population approach was implemented in healthy volunteers. The reference value obtained by this approach is of great importance in order to simulate appropriate doses and to evaluate potential interactions between concomitant drugs. Furthermore, it presents a baseline pharmacokinetic model in healthy volunteers, suitable for in silico clinical trial simulations in order to design informative future clinical trials.

The final model in the present study was a one-compartment model, which adequately described the four sequences simultaneously. Adding a second compartment decreased the OFV significantly (*p* < 0.05). However, this resulted in a decreased precision in the parameter estimates and an unreasonable estimate of the terminal elimination half-life (127 h) [17, 19, 35, 36]. Therefore, a one-compartment model was retained. Sidhu et al. [35] reported on a study in 23 children and 31 adults with uncomplicated falciparum malaria receiving a 5-day oral treatment of artemisinin. Two capillary blood samples were collected after the first dose and one sample (from 30 % of the patients) after the last dose on day five. This study also concluded that a one-compartment model described the data adequately. Batty et al. [36] investigated the exposure of artemisinin in paediatric patients after a combination treatment of artemisinin–naphtoquine in three groups with different doses. Rich data were collected for 48 h resulting in a two-compartment model as the best fit for the data. The implementation of this more complex model is possibly due to the longer sampling time compared to the present study.

Multiple absorption peaks and a large variability during the absorption phase were observed in some subjects in all treatment sequences which is in agreement with previously published data [35, 50]. In contrast to Sidhu et al. and Batty et al. a flexible transit compartment absorption model was successfully applied for the absorption profile. In both previous studies a first-order absorption was found to be the best fit to the observed data, with a lag time in the paediatric study. The different absorption models could be explained by more frequent sampling in the absorption phase in this study. Overall, parameter estimates of the final model were estimated with a high precision in this study due to a highly informative sampling schedule with rich data in both the absorption and the elimination phase. Parameter estimates in this work in healthy volunteers were also in agreement with those reported in patients by Sidhu et al. after taking into account the differences in bodyweight (allometrically scaled with a fixed exponent [$CL_{study1} = CL_{study2} *\left( {BW_{study1} /BW_{study2} } \right)^{0.75}$] for CL) [35, 44, 51].

The data in the present study was previously used in a non-compartmental analysis by Hien et al. [23]. In that analysis, the two different formulations were concluded to not fulfill the criteria for bioequivalence due to large variability, according to the FDA guidelines [52]. However, the clinical impact was considered to be negligible [23]. The full covariate population modelling approach used in the present study showed similar results with no absorption-related pharmacokinetic differences between the reference formulation and the micronized powder formulation. The effect of the combination with piperaquine was also investigated using the same approach and showed no effect on the pharmacokinetics of artemisinin. A 3-week washout period between dosing occasions was incorporated in this study. This is enough to clear systemic artemisinin concentrations (i.e. >5 artemisinin half-lives). On the other hand, piperaquine half-life is reported to be more than 25 days [53, 54] which could be a possible limitation in the protocol and was therefore taken into consideration when evaluating the results. However, no apparent effect was found on artemisinin in actual co-administration with piperaquine, indicating that the low concentrations of piperaquine remaining after a 3-week washout period should not influence the pharmacokinetic properties of artemisinin. Furthermore, the risk of over predicting the influence of piperaquine on artemisinin pharmacokinetics (type I error) was assumed to be larger than the risk of not finding an effect (type II error) and therefore the limitation in the study protocol was considered negligible. The study design with two different dose sizes for the conventional formulation was due to earlier findings suggesting dose-dependent artemisinin pharmacokinetics [16, 55]. However, dose differences did not change the relative bioavailability although the mean transit-time increased substantially at higher doses in the present study. Possible explanations for this prolonged absorption could be low solubility or elongated dissolution of the drug powder. It has previously been shown that artemisinin has a high permeability via passive diffusion, therefore, transportation across the intestinal membranes is not likely to be a rate-limiting step increasing mean transit-time [32].

The developed population pharmacokinetic model showed good predictive performance (Fig. 3) and is, therefore, suitable for population based simulations and clinical trial design. Mean concentration–time profiles for three groups, healthy volunteers, adult patients and paediatric patients, were simulated after receiving identical dosages (weight adjusted) of artemisinin as in the present study (Fig. 5). The time to reach maximum concentration occurs at almost the same time in all three groups, although adult patients have a lower maximum concentration compared to healthy volunteers and pediatric patients. The absorption models differ between healthy volunteers and patients, most likely due to sparse sampling in the absorption phase in the two patient studies. The exposure to artemisinin in children is unexpectedly higher than that in adult patients and similar to healthy volunteers. The higher exposure could be explained by a substantially higher dose in the study by Batty et al. [36] which could possibly result in a saturated elimination. This indicates that dose adjustment by allometric scaling would be preferable to achieve comparable exposure as in healthy volunteers. This needs to be studied further for a firm paediatric dosing recommendation.

## Conclusions

In conclusion, this is the first population pharmacokinetic characterization of artemisinin in healthy volunteers. The highly informative dense sampling resulted in a one-compartment disposition model with a transit absorption model. Increasing the dose resulted in a significant increase in the mean transit-time but the micronized formulation or concomitant piperaquine administration did not affect the pharmacokinetic properties of artemisinin. The results from the traditional model-independent bioequivalence approach were in agreement with results obtained from the modelling approach, based on the limited data from only one clinical study. The developed final model may be an important tool to investigate new dosing regimens in silico and to be implemented in clinical trial simulations for informative design of future clinical trials.
